# Complete Genome Sequence of Porcine Encephalomyocarditis Virus from an Aardvark in China

**DOI:** 10.1128/genomeA.00017-14

**Published:** 2014-02-13

**Authors:** Hongtao Chang, Lu Chen, Jun Zhao, Xinwei Wang, Xia Yang, Huixia Yao, Chuanqing Wang

**Affiliations:** College of Animal Science and Veterinary Medicine, Henan Agricultural University, Zhengzhou, Henan Province, China

## Abstract

A strain of encephalomyocarditis virus, HNXX13, was isolated from an aardvark in central China. The complete genome was sequenced and analyzed, and phylogenetic analysis suggests that HNXX13 belongs to encephalomyocarditis virus group 1.

## GENOME ANNOUNCEMENT

Strains of encephalomyocarditis virus (EMCV), a positive-sense, single-stranded RNA virus belonging to the genus *Cardiovirus* of the family *Picornaviridae*, are a group of closely related virus strains with a wide host range (1). Infection with EMCV is associated with sporadic cases and outbreaks of myocarditis and encephalitis in domestic pigs, numerous species of nonhuman primates, and other mammalian species (2). Using Chinese serological and etiological methods, it was confirmed that EMCV infection has occurred on many pig farms in recent years (3).

In this study, EMCV strain HNXX13 was isolated from heart and brain tissue samples from an aardvark that exhibited clinical signs of myocarditis in Henan province, China, in 2013. The whole open reading frame (ORF) of EMCV was amplified and sequenced using six pairs of specific primers. Both 5′ and 3′ untranslated regions (UTRs) were amplified by the 5′ and 3′ full RACE core sets (TaKaRa Biotechnology Company, Dalian, China). The PCR products were gel purified, cloned into the pMD19-T vector, and sequenced with an Applied Biosystems (ABI) 3730xl DNA analyzer. Sequence assembly was carried out using the SeqMan program of the DNAStar software. The full-genome sequencing and assembly of EMCV HNXX13 generated a sequence of 7,725 bp in length, with a 5′ UTR of 708 nucleotides, a 3′ UTR of 138 nucleotides, and a large ORF of 6,879 nucleotides. The whole ORF of HNXX13 encodes 11 proteins that are similar to those reported previously for other EMCV strains, including VP1 to VP4, 2A to 2C, 3A to 3D, and a virus-leading protein (L protein) (3–5).

Phylogenetic analysis and multiple sequence alignment according to the complete nucleotide sequence revealed that EMCV isolates cluster into two groups (groups 1 and 2), with two subclusters within group 1 (1a and 1b), and that HNXX13 belongs to group 1a with other Chinese EMCV strains. Compared to other previously reported EMCV strains, the complete genome sequence of HNXX13 exhibits different nucleotide sequence identities: 81.0% to 99.9% with foreign and domestic EMCV strains from different animals and 99.6% with Chinese strains (NJ08, GX0601, and GXLC). Multiple sequence alignment based on the ORF was completed by means of the DNAStar software, using other available strains from the GenBank nucleotide database. A comparison of the amino acid sequences of VP1 from HNXX13 with those from other Chinese isolates showed 3 amino acid mutations of fragments containing hydrophilic regions: K7R, A13T, and G63Q. The relationship between the genomic information and pathogenicity needs to be further investigated.

### Nucleotide sequence accession number.

The genome sequence of EMCV strain HNXX13 has been deposited in GenBank under the accession no. KF771002.
